# Vortex radiation from a single emitter in a chiral plasmonic nanocavity

**DOI:** 10.1515/nanoph-2021-0743

**Published:** 2022-02-25

**Authors:** Xing-Yuan Wang, Hua-Zhou Chen, Suo Wang, Li Ge, Shuang Zhang, Ren-Min Ma

**Affiliations:** State Key Lab for Mesoscopic Physics and Frontiers Science Center for Nano-optoelectronics, School of Physics, Peking University, Beijing, China; Department of Physics and Astronomy, College of Staten Island, CUNY, Staten Island, New York, USA; Graduate Center, CUNY, New York, USA; Department of Physics, University of Hong Kong, Hong Kong, China; School of Physics and Astronomy, University of Birmingham, Birmingham B15 2TT, UK

**Keywords:** anomalous spontaneous emission, exceptional point, nanocavity, optical vortex, parity-time symmetry, single emitter

## Abstract

Manipulating single emitter radiation is essential for quantum information science. Significant progress has been made in enhancing the radiation efficiency and directivity by coupling quantum emitters with microcavities and plasmonic antennas. However, there has been a great challenge to generate complex radiation patterns such as vortex beam from a single emitter. Here, we report a chiral plasmonic nanocavity, which provides a strong local chiral vacuum field at an exceptional point. We show that a single linear dipole emitter embedded in the nanocavity will radiate to vortex beam via anomalous spontaneous emission with a Purcell enhancement factor up to ∼1000. Our scheme provides a new field manipulation method for chiral quantum optics and vortex lasers at the nanoscale.

## 1 Introduction

In quantum information science, one of the prime tasks is to generate single photon states on demand from a single quantum emitter, such as an atom, a quantum dot or a nitrogen-vacancy center in diamond [1], [2], [3], [4]. Cavity quantum electrodynamics (QED), which studies the interaction between a quantum emitter and cavity modes, has played a central role in this pursuit of developing practical sources of quantum states of light [1, 5]. For instance, a single quantum dot emitter coupled to a micropillar cavity has been employed in the recent demonstration of quantum boson-sampling machines with superior performance [6]. Another platform for solid-state cavity QED, namely plasmonic waveguides and cavities, have recently attracted growing interest in modifying radiation efficiency and directivity of single quantum emitters, where plasmonic effect with strong field localization enhances light–matter interaction significantly [2, 3, 7].

In the meanwhile, phase singularities or optical vortices have also received an ever increasing amount of attention from the optics community [8, 9]. Most noticeably, devices that emit individual photons carrying orbital angular momentum (OAM) provide an exciting platform for using OAM in quantum information science, as they allow additional encoding on the single photon level [10, 11]. Moreover, a multistate OAM system can be combined with spin angular momentum (SAM) or other degrees of freedom to form hyper entanglement or hybrid entanglement [12, 13], which can significantly improve quantum computation, quantum communication, and quantum cryptography. As reported recently [13], a single photon encoded with both SAM and OAM has been utilized for quantum teleportation of composite states.

Notwithstanding the fast development of cavity QED in preparing single photon states, modulating the radiation pattern of a single emitter into a vortex beam with controllable topological charge remains a formidable task. While one can introduce chirality to the scattering light field of a nanoparticle or a nanoslit using circularly polarized light illumination in the classical regime, it is much more sophisticated to control the chirality of the radiation field of a single emitter in the quantum regime, where the Zeeman effect has to be introduced in the system, for instance [14], [15], [16], [17]. A promising approach that has been demonstrated in the emergent chiral quantum optics employs spin-momentum locking, i.e., placing a circularly polarized emitter in the vicinity of optical waveguides or cavities [4, 15, 18, 19].

An alternative approach to introduce chiral light–matter interaction puts more emphasis on the photonic environment, which allows only unidirectional wave propagation. A novel class of chiral photonic structures are introduced using parity-time (PT) symmetry [20] and its resultant non-Hermitian properties [21]. PT symmetry requires an effectively balanced arrangement of optical gain and loss [22], [23], [24], [25], [26], and unidirectional reflectionless transmissions in a straight waveguide [27, 28] have been shown to be the result of a generalized flux conservation relation [29]. When wrapped into a ring, which would have two traveling-wave modes with opposite OAMs in the absence of PT modulation, a single coalesced OAM mode emerges as the result of an exceptional point of the system [30]. Such an optical exceptional point has been employed to construct chiral optical devices, including single mode lasers and vortex lasers [31], [32], [33], [34]. More interestingly, at an exceptional point, an emitter can display the opposite handedness to the coalesced eigenstate of the system [35]. Such anomalous spontaneous emission effect breaks the conventional wisdom that an emitter radiates into and interacts with eigenstates of the photonic environment, and has been verified experimentally in microwave and acoustic systems [35].

Here, for the first time, we report a nanoscale vortex emitter based on a chiral plasmonic nanocavity (CPN). We introduce PT symmetric refractive index modulation into a plasmonic nanocavity which results in a strong local vacuum field at the exceptional point of the system. The mode volume and quality factor of the CPN are 0.24 × ${\left(\frac{\lambda }{2n_{\text{eff}}}\right)}^{3}$ and 480 respectively, which lead to an embedded linear dipole emitter with high spontaneous emission coupling factor approaching to 1 and Purcell enhancement factor approaching to 1000. The chirality of the radiation field of the emitter embedded inside the CPN is position dependent. It can be the same with the coalesced eigenmode, which can be treated as a normal spontaneous emission process. It can also be opposite to the coalesced eigenmode, which is via the aforementioned anomalous spontaneous emission effect. Our work enriches the interesting physics of an exceptional point in the quantum regime, and provides a new method for developing chiral quantum optics and vortex lasers at nanoscale.

## 2 Results and discussion

**Figure 1:**
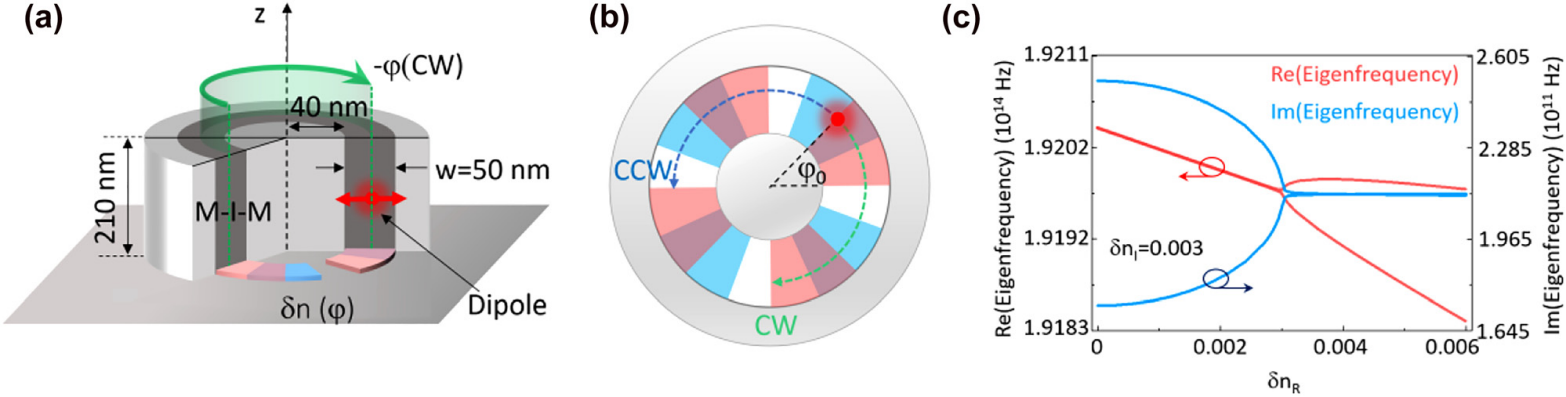
Chiral plasmonic nanocavity. (a) Schematic of a chiral plasmonic nanocavity (CPN). M-I-M represents metal-insulator-metal. (b) PT symmetric modulation δn(φ) in CPN. δn(φ) is divided into 2l periods for WGMs with orbital angular momentum *l*. Each period of δn(φ) consists of four angularly equidistant parts of $\delta n_{\text{R}}$ , $\delta n_{\text{R}}+\delta n_{\text{I}}i$ , $\delta n_{\text{I}}i$ and 0 arranging in counterclockwise direction, where $\delta n_{\text{R}}$ and $\delta n_{\text{I}}$ denote the real part and imaginary part of δn(φ) respectively. $\varphi_{0}$ is determined by the relative position of the linear dipole emitter to the refractive index modulation. (c) The evolution of the eigenfrequency of the CPN as a function of $\delta n_{\text{R}}$ , with a fixed $\delta n_{\text{I}}=0.003$ . The eigenfrequencies coalesce when $\delta n_{\text{I}}=\delta n_{\text{R}}=0.003$ .

Figure 1(a) illustrates the design of a CPN operating at an exceptional point, which is a ring resonator with a metal-insulator-metal coaxial geometry. The bottom of the insulator ring is encapsulated by silver and with a patterned layer to introduce PT symmetric refractive index modulation (Figure 1(b)). A single linear dipole emitter is embedded inside the insulator region, and it is at resonance with a pair of whispering-gallery modes (WGMs). The electric field in the CPN can be written as: $E\left(\rho ,\varphi ,z\right)=U\left(\rho ,z\right)\left[a_{\text{CCW}}\left(t\right)e^{il\varphi }+a_{\text{CW}}\left(t\right)e^{-il\varphi }\right]e^{-i\omega t}$ , where U(ρ,z) is the transverse mode profile for the clockwise (CW) and counterclockwise (CCW) modes at *ρz* plane. To describe our single linear dipole emitter embedded CPN, we formulate the coupled mode equations below by including (i) the coupling between the single emitter and the two degenerated counter-propagating WGMs; and (ii) the coupling between these two WGMs (electric field):

(1) $$\frac{d}{dt}a_{\text{CW}}=-i\omega a_{\text{CW}}-\gamma_{\text{tot}}a_{\text{CW}}+\chi_{\text{ab}}a_{\text{CCW}}+\epsilon s,$$

$$\frac{d}{dt}a_{\text{CCW}}=-i\omega a_{\text{CCW}}-\gamma_{\text{tot}}a_{\text{CCW}}+\chi_{\text{ba}}a_{\text{CW}}+\epsilon s.$$

Here $a_{\text{CW}}$ ( $a_{\text{CCW}}$ ) is the amplitude of the CW (CCW) mode, ω is the traveling WGM resonance frequency , $\gamma_{\text{tot}}$ is the total loss rate of the cavity, and $\chi_{\text{ab}}$ ( $\chi_{\text{ba}}$ ) is the coupling coefficient from the CCW (CW) mode to the CW (CCW) mode. The linear dipole emitter appears as a driving term in Eq. (1), represented by the instantaneous radiation amplitude s and the coupling coefficient ϵ to the cavity fields (Note S1).

Under the condition of s=0 (no source inside the cavity), we can solve the (complex) resonant frequencies of the CPN from Eq. (1) as

(2) $$\begin{array}{c} \Omega_{\pm }=\omega -i\gamma_{\text{tot}}\pm i\sqrt{\chi_{\text{ab}}\chi_{\text{ba}}} \end{array}$$

and the two corresponding eigenstates are given by

(3) $$\begin{array}{c} \left(\begin{array}{c} \alpha_{\text{CW}}\\ \alpha_{\text{CCW}} \end{array}\right)=\left(\begin{array}{c} \pm \sqrt{\chi_{\text{ab}}}\\ \sqrt{\chi_{\text{ba}}} \end{array}\right). \end{array}$$

Clearly, the exceptional point of a CPN can be reached when one of the two coupling coefficients ( $\chi_{\text{ab}},$ $\chi_{\text{ba}}$ ) equals zero, where the two eigenmodes given by Eq. (3) coalesce into a single chiral mode.

Instead of implementing the sinusoid PT-symmetric refractive index modulation, we approximate it by a square waveform (Figure 1(b)). The corresponding coupling coefficients are proportional to the Fourier transform coefficients of δn(φ) with angular momentum ±2l , which can be expressed as

(4) $$\begin{array}{c} \chi_{\text{ab},\text{ba}}=\kappa \left(\delta n_{\text{I}}\mp \delta n_{\text{R}}\right)e^{\mp i2l\varphi_{0}}. \end{array}$$

Here κ is a constant and $e^{i2l\varphi_{0}}$ is a phase factor determined by the position of the dipole emitter (Note S1). $\varphi_{0}$ is determined by the relative position of the dipole to the grating (Note S1). Clearly, the backscattering is unidirectional when $\delta n_{\text{I}}$ equals $\delta n_{\text{R}}$ , with $\chi_{\text{ab}}=0$ but $\chi_{\text{ba}}\neq 0$ . Consequently, the eigenstates coalesce to one chiral CCW (right-handed) mode.

Now we consider the radiation field of a single linear dipole emitter embedded in the CPN at the exceptional point. At resonant frequency, the amplitude ratio of the CCW and CW waves in the radiation field can be calculated using Eq. (1), and it is given by

(5) $$\begin{array}{c} \frac{a_{\text{CCW}}}{a_{\text{CW}}}=1+\frac{\chi_{\text{ba}}}{\gamma_{\text{tot}}}=1+\frac{2\kappa \delta n_{\text{I}}}{\gamma_{\text{tot}}}e^{i2l\varphi_{0}} \end{array}$$

in the steady state.

**Figure 2:**
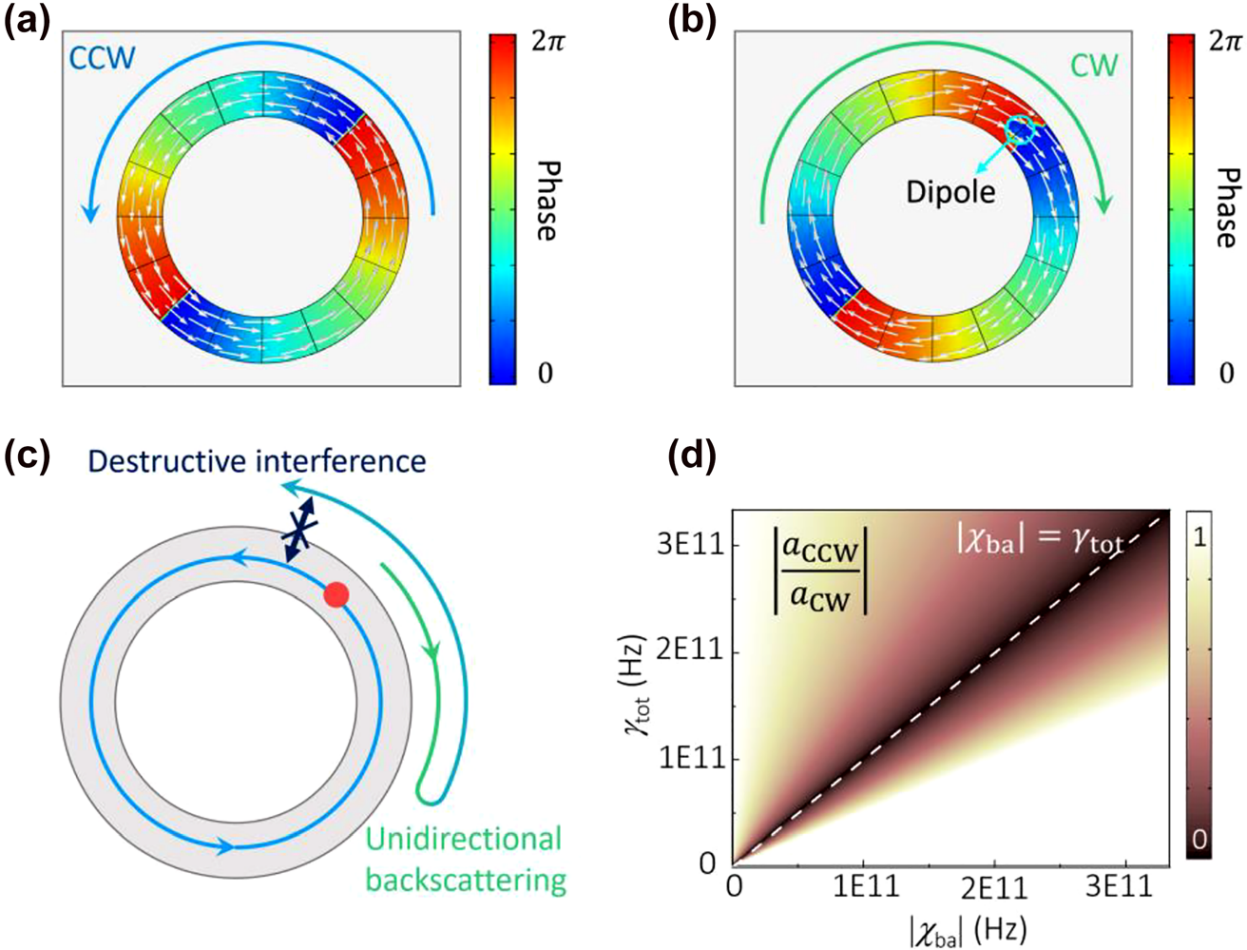
Anomalous spontaneous emission at exceptional point. (a) and (b) Phase distribution of the radial electric field ${\text{E}}_{\rho }$ of the coalesced eigenmode (a) and the single emitter excited mode (b). The white arrows denote Poynting vectors. (c) The anomalous spontaneous emission effect is due to the completely destructive interference between the directly radiated CCW wave and the backscattered CCW wave. (d) Amplitude ratio between the CCW and CW waves in the radiation field. Anomalous spontaneous emission effect occurs when $\chi_{\text{ba}}=-\gamma_{\text{tot}}$ .

We can see that the chirality of the single emitter radiation field inside the CPN depends on the location of the dipole emitter. At $e^{i2l\varphi_{0}}=1$ , the ratio of $\frac{a_{\text{CCW}}}{a_{\text{CW}}}$ reaches its maximum, and the single emitter radiates to the coalesced right-handed eigenmode. However, at $e^{i2l\varphi_{0}}=-1$ , the ratio of $\frac{a_{\text{CCW}}}{a_{\text{CW}}}$ vanishes at $\chi_{\text{ba}}=-\gamma_{\text{tot}}$ , and the single emitter radiates only to the missing dimension of the left-handed mode at the exceptional point via anomalous spontaneous emission effect (Figure 2) [35].

In the following, we focus on the left-handed mode excited by the single emitter via anomalous spontaneous emission. Such a chiral radiation field will radiate to the free space in the form of vortex beam, because of the similarity between the cavity field and the free space vortex beams. Both of them consist of Bessel functions in the radial direction and a phase factor of $e^{i\left(l\varphi +k_{z}z\right)}$ that couples the azimuthal and vertical directions. In the vertical direction *z*, the excited chiral cavity mode is a standing wave consisting both negative and positive *z*-momentum. As the CPN is half encapsulated, the only outgoing wave in the positive *z* direction leads to a vortex radiation to free space.

Based on the principle discussed above, we design two vortex emitters with distinct material systems and operation wavelengths and then verify them via full wave simulations. The first one is designed to operate at 1550 nm, where the material system of an InAs quantum dot embedded in InP is adopted [36]. The second one is designed to operate at 900 nm, where the material system of InAs quantum dot embedded in GaAs is chosen [36]. In the following, we show the result of the first design as an example, while the other one is presented in Figure S1.

In the design, the height of the CPN is 210 nm and the width of the insulator ring is 50 nm. The inner diameter of the insulator ring is varied by tens of nanometer for the desired OAM in the vortex radiation. The dipole is positioned at $\varphi_{0}=\frac{\pi }{2l}$ as required by the condition $\chi_{\text{ba}}=-\gamma_{\text{tot}}$ , and the refractive index modulation is set to $\delta n_{\text{I}}=\delta n_{\text{R}}=0.003$ (Note S2 and Figure S2). We note that the dimensions of the CPN ensure that only one fundamental symmetric plasmonic mode is supported, which is necessarily to achieve a near unity spontaneous emission coupling ( β ) factor. The strongly confined electromagnetic field inside the CPN at resonance leads to a high Purcell factor ( $F_{\text{p}}$ ), which will be discussed in detail below (Figure S3). We note that for practical quantum optics application of a single emitter, β↦1 and $F_{\text{p}}\gg 1$ are necessary for both high collection efficiency and the suppression of non-radiative emissions.

**Figure 3:**
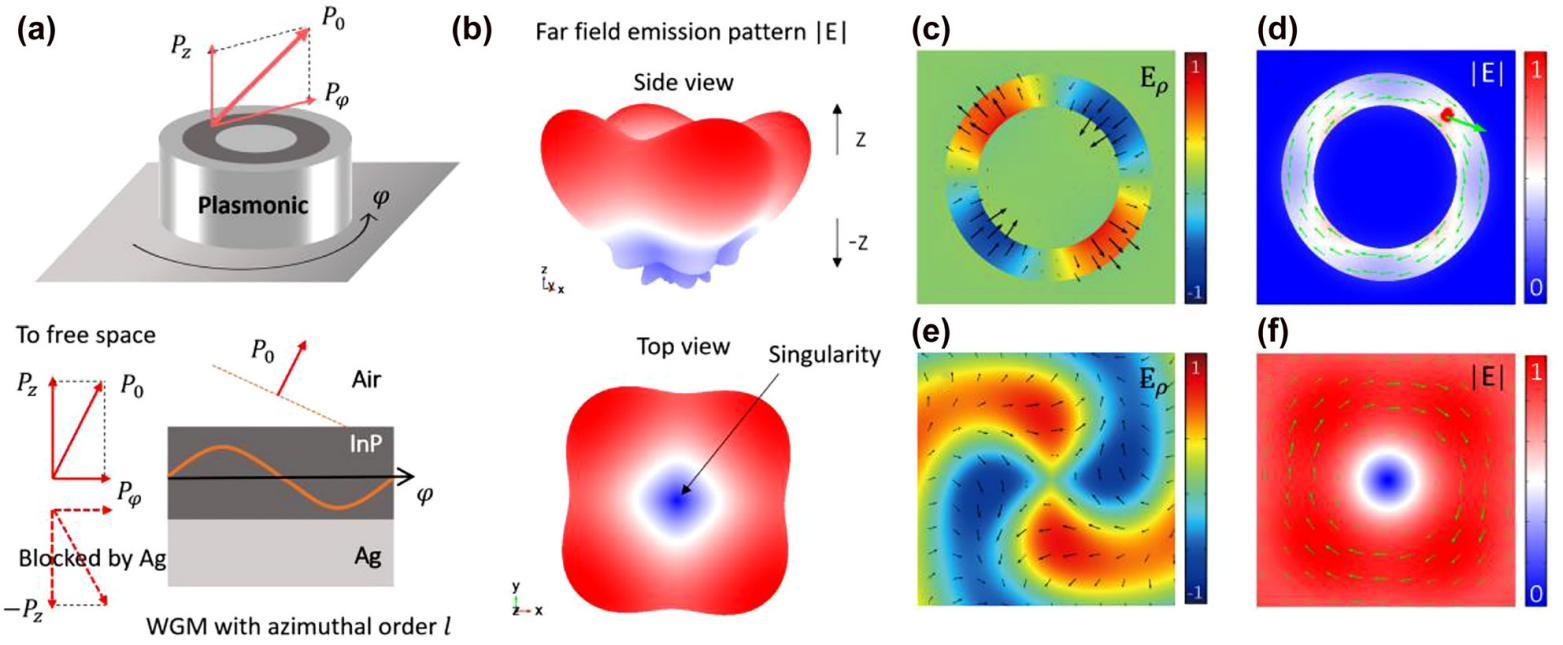
Single emitter vortex radiation at telecommunication wavelength. (a) The device is designed to operate at 1550 nm, where an InAs quantum dot is embedded in the middle InP ring (dark) region. Chiral cavity mode will radiate to free space as vortex beam from the open facet of the nanocavity. $P_{0}$ , $P_{z}$ , and $P_{\varphi }$ represent the total momentum and its *z* and azimuthal components, respectively. (b) Simulated perspective view (upper panel) and top view (bottom panel) of the far field radiation pattern of the device. (c)–(f) $E_{\rho }$ and |**E**| of the single emitter excited field inside the cavity ((c) and (d)) and at a height of 1550 nm above the cavity ((e) and (f)). In (c)–(e), the black and green arrows denote polarizations and Poynting vectors, respectively. In (f), the green arrows denote azimuthal component of Poynting vector.

Figure 3(b) shows the simulated far field pattern of the dipole radiation, where most energy is emanated to free space from the upper facet of the cavity (Figure 3(a)). To show its vortex nature, in Figure 3(c) and (d) we plot $E_{\rho }$ and |**E**| of the radiation field inside the cavity. $E_{\rho }$ is the dominant field here and it displays features of a radially polarized WGM with l=−2 (Figure 3(c)); the uniform |**E**| field in the azimuthal direction and the circulating Poynting vector shown in Figure 3(d) also indicate that the excited field is indeed a traveling chiral WGM. Furthermore, we plot $E_{\rho }$ and |**E**| at a height of 1550 nm above the cavity in Figure 3(e) and (f). The spiral pattern of $E_{\rho }$ reveals a phase factor of $e^{i2\varphi }$ (Figure 3(e)), and the undefined phase at the center indicates a topological phase singularity on the beam axis (Figure 3(b), Figure 3(e) and Figure S4). In addition, the Poynting vector of the emission beam shares the same circulating feature as the field inside the cavity (Figure 3(f)). These results unambiguously confirm that the CPN twists the single linear dipole emission into a vortex beam with a topological charge of −2 . By tuning the azimuthal order of the WGM, we confirmed that a linear dipole can also generate vortex emission with other well defined topological charges ( l=−1 and −3 are shown in Figure S5).

As we have mentioned above, the coupling between the CW and CCW fields inside the cavity depends on $\varphi_{0}$ and so does the chirality of the dipole radiation, which can be defined quantitatively as [4]

(6) $$\begin{array}{c} \alpha =1-\frac{\min\left[\beta_{\text{CW}},\beta_{\text{CCW}}\right]}{\max\left[\beta_{\text{CW}},\beta_{\text{CCW}}\right]}. \end{array}$$

**Figure 4:**
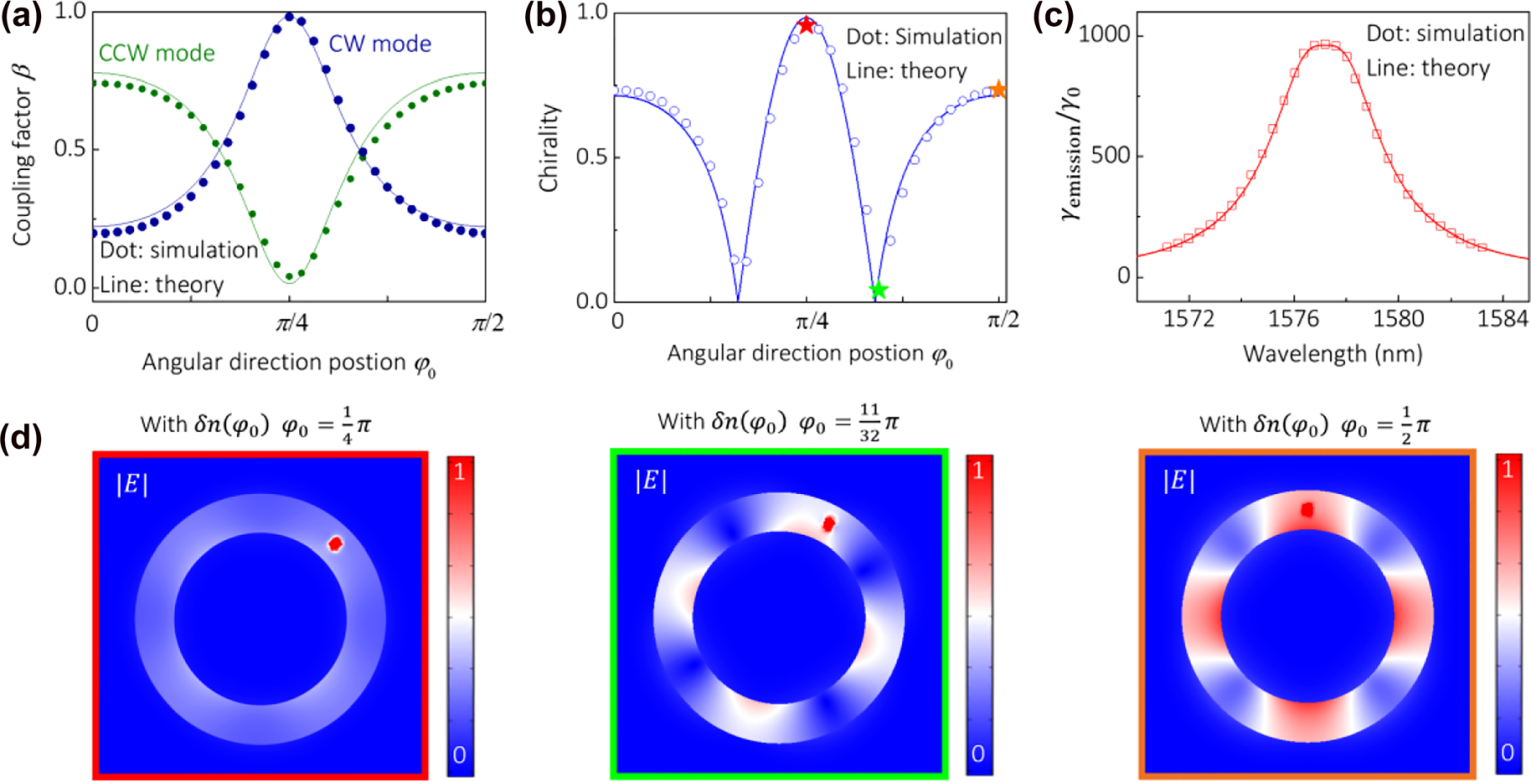
Chirality and radiation rate enhancement of a single emitter inside the CPN. (a) $\beta_{\text{CW}}$ and $\beta_{\text{CCW}}$ as a function of the dipole position $\varphi_{0}$ at resonance. (b) Chirality of the single emitter radiation at resonance as a function of its position $\varphi_{0}$ . (c) Radiation rate acceleration factor $\gamma_{\text{emission}}/\gamma_{0}$ at varied wavelength under the condition that $\varphi_{0}=\pi /4$ . In (a)–(c), dots and solid lines are obtained by full wave simulation and coupled mode theory respectively. (d) The electric field excited by a single emitter located at different azimuthal positions.

Here $\beta_{\text{CW}\left(\text{CCW}\right)}$ is the β factor of CW (CCW) field (Note S3 and Figure S6). Figure 4(a) shows the simulated β factors of the dipole radiation at resonance as a function of its position $\varphi_{0}$ . The total β factor of the two |l|=2 chiral modes ( $\beta_{\text{CW}}+\beta_{\text{CCW}}$ ) approaches unity, as a result of the large Purcell factor and the large free spectrum range of our CPN. Figure 4(b) shows the simulated chirality of the dipole radiation, and its maximum is reached at $\varphi_{0}=\pi /4$ , where a traveling chiral CW mode is excited with $\beta_{\text{CW}}\approx 0.9813$ . In Figure 4(d), we show the electric field distribution excited by the dipole at different azimuthal positions. It can be clearly seen that the ratio of the CW and CCW field can be controlled by tuning the azimuthal position of the dipole. The simulation results are in very good agreement with the numerical calculation from the coupled mode theory (Solid line in Figure 4(a) and (b)).

The spontaneous emission rate ( γ , γ=1/τ , τ : emission lifetime) can be increased by spatial and spectral confinement of the optical field, known as the Purcell effect [37]. A high emission rate is crucial for a quantum emitter with large quantum efficiency and emission rate, and it also suppresses the blinking of a quantum emitter. The Purcell enhancement factor ( $F_{\text{P}}$ ) is proportional to $Q/V_{\text{mode}}$ , where Q and $V_{\text{mode}}$ are the quality factor and mode volume of a cavity, respectively. Our CPN has an extremely small $V_{\text{mode}}$ of 0.24 × ${\left(\frac{\lambda }{2n_{\text{eff}}}\right)}^{3}$ and a mediate Q of 480 (See method). Here we calculate the radiative decay rate acceleration factor by the cavity which is defined as $\gamma_{\text{emission}}/\gamma_{0}$ , where $\gamma_{\text{emission}}$ and $\gamma_{0}$ are the radiative decay rates of a dipole emitter in the nanocavity and free space, respectively (See method). Figure 4(c) shows $\gamma_{\text{emission}}/\gamma_{0}$ at varied wavelength under the condition of $\varphi_{0}=\pi /4$ . At zero detuning, the radiative decay rate is accelerated by 965 times. We also calculate the $\gamma_{\text{emission}}/\gamma_{0}$ by the coupled mode theory (red solid line), which matches well the simulation result. Preliminary results of this article were posted on arXiv [38] and reported in conference presentations [39].

## 3 Conclusions

In summary, we report strong chiral vacuum field constructed in a plasmonic nanocavity with parity-time symmetry refractive index modulation. The nanocavity has a small mode volume of 0.24 × ${\left(\frac{\lambda }{2n_{\text{eff}}}\right)}^{3}$ and a high quality factor of 480. The strong localized vacuum field enhances the light–matter interaction inside the cavity substantially, which enables a near unity spontaneous emission coupling factor and a ∼1000 Purcell factor for an embedded single emitter. More importantly, the radiation field of an embedded single emitter is vortex beam carrying orbital angular momentum, where the topological charge of the vortex beam can be tuned. The chiral plasmonic nanocavity can also be used to construct vortex nanolaser with low threshold [40, 41].

## 4 Methods

### 4.1 Full wave numerical simulations

The simulations are calculated by the finite element electromagnetic solver (COMSOL) with tetragonal meshing and scattering boundary conditions. In 2D simulations, the maximum and minimum element size of different regions are 15⁄*n* nm and 0.15⁄*n* nm respectively, where *n* is the real part of the refractive index in different regions. The maximum element growth rate is 1.1, the curvature factor is 0.2, and the resolution of narrow regions is 1. We use the direct MUMPS with a convergence relative tolerance of $10^{-6}$ . In 3D simulations, the general maximum element size is 144⁄*n* nm, and the general minimum element size is 1.44⁄*n* nm; We use finer mesh in the vicinity of the dielectric (InP)/metal (Ag) interface, where the energy of plasmonic mode is mainly confined. The maximum element size in the InP/InGaAsP region is 7.7 nm. The maximum element size in the metal region (in the vicinity (100 nm) of the cavity) is 10.3 nm. And the maximum element growth rate is 1.3. The curvature factor and resolution of narrow regions are the same as the 2D simulations. We use the direct MUMPS with a convergence relative tolerance of $10^{-3}$ . In the dipole excited field simulations, since the linewidth of single emitters (∼0.01 nm) can be much narrower than the linewidth of the cavity mode, we set the linewidth of dipole source as a delta function. In the simulation, we consider the condition that the temperature is set to be 4.5 K to reduce the metal loss. The refractive index of the material is set as follows: $n_{\text{InP}}=3.0806$ , $n_{\text{Cr}}=3.6683+4.18i$ , $n_{\text{Ge}}=4.275+0.00567i$ , and $n_{\text{Ag}}=0.0014+10.9741i$ . $n_{\text{InGaAsP}}=3.34$ for InGaAsP, $n_{{\text{Al}}_{2}{\text{O}}_{3}}=1.6214+0.00008i$ for ${\text{Al}}_{2}{\text{O}}_{3}$ .

### 4.2 Numerical calculations of *Q* values, mode volumes and Purcell enhancement

The *Q* value is calculated from the formula $Q=f_{\text{r}}/\Delta f$ , where the $f_{\text{r}}$ is the resonance frequency and Δf is the full width at half maximum of the resonance spectrum. The mode volume is calculated from $V_{\text{m}}=\frac{W_{\text{total}}}{\text{max}\left[W\left(r\right)\right]}$ , where $W_{\text{total}}$ is the total mode energy integrated over the entire space, i.e., $W_{\text{total}}=∭W\left(r\right)d^{3}r$ . W(r) is the local energy density $W\left(r\right)=\frac{1}{2}\left(\text{Re}\left[\frac{\text{d}\left(\omega \varepsilon \right)}{\text{d}\omega }\right]{\left|E\left(r\right)\right|}^{2}+\mu {\left|H\left(r\right)\right|}^{2}\right)$ . The peak energy density max[W(r)] is found by comparing all the energy density in the entire simulation regions. Here, ε and μ are permittivity and permeability of the materials, respectively. The dispersion item $\omega \frac{\text{d}\varepsilon }{\text{d}\omega }$ of Ag is 284.1. The radiative decay rate enhancement is calculated by $\gamma_{\text{emission}}/\gamma_{0}=\frac{P_{\text{cavity}}}{P_{\text{free space}}}$ , where $P_{\text{cavity}}$ and $P_{\text{free space}}$ are the radiated powers of the dipole when the dipole is placed in the cavity and free space, respectively.

## Supplementary Material

Supplementary Material
