# Supplementary material for: Vortex radiation from a single emitter in a chiral plasmonic nanocavity
Source: Nanophotonics. 2022 Feb 25;11(9):1905–11. doi: 10.1515/nanoph-2021-0743 (PMC11501179; doi:10.1515/nanoph-2021-0743)
Supplement: Supplementary file 1 — Supplementary Material [file j_nanoph-2021-0743_suppl.pdf]

Supporting Information

Vortex radiation from a single emitter in a chiral plasmonic nanocavity

Xing-Yuan Wang[1,6], Hua-Zhou Chen[1], Suo Wang[1], Li Ge[2,3], Shuang Zhang[4,5], Ren-Min Ma[1*]

[1] State Key Lab for Mesoscopic Physics and Frontiers Science Center for Nano-optoelectronics, School of Physics, Peking University, Beijing, China

[2] Department of Physics and Astronomy, College of Staten Island, CUNY, Staten Island, NY, USA.

[3] Graduate Center, CUNY, New York, USA.

[4] Department of Physics, University of Hong Kong, Hong Kong, China.

[5] School of Physics and Astronomy, University of Birmingham, Birmingham, B15 2TT, UK

[6] Current address: College of Mathematics and Physics, Beijing University of Chemical Technology, Beijing, China

[*]Correspondence should be addressed to renminma@pku.edu.cn.

Content

Note S1: Derivation of coupled mode equations 2

Note S2: Detailed full wave simulation description. 5

Fig. S1: Single emitter vortex radiation at 900 nm 5

Fig. S2: Effective refractive index of the cavity modes 7

Fig. S3: Strong field confinement of the cavity mode 8

Fig. S4: Radiation field characterization of the cavity modes 9

Fig. S5: Vortex beam emission with varied topological charges 10

Note S3: Numerical calculation of the spontaneous emission coupling factor 11

Fig. S6: The calculation of spontaneous emission coupling factor 11

Note S1: Derivation of coupled mode equations

In passive PT system, the refractive index modulation will introduce loss to the system. This modulation loss γ_{mod} and the coupling coefficients $\chi_{\text{ab(ba)}}$ are proportional to the Fourier transformation coefficients C_0 , C_{-2l} and C_{2l} of the PT symmetric modulation at $0, -2l, 2l,$ respectively. For PT symmetric modulation shown in Fig. 1b in the main text (square waveform of the index modulation), if we define the proportionality factor as $\pi\kappa$, we have:

$$\gamma_{\text{mod}} = \pi\kappa C_0 = \frac{\pi}{2}\kappa\delta n_{\text{I}}$$

$$\chi_{\text{ab(ba)}} = \pi\kappa C_{\mp 2l} = \kappa(\delta n_{\text{I}} \mp \delta n_{\text{R}}) \tag{1.1}$$

Note that the real part of Fourier transformation coefficients at 0 indicates frequency detuning and have equal impact on the two modes, and thus it is ignored here. When δn_{I} equals δn_{R}, backscattered fields by real and imaginary parts of refractive index modulation interfered constructively in one direction but destructively in the other direction, leading to unidirectional backscattering. At $\delta n_{\text{I}} = \delta n_{\text{R}}$, the loss induced by the modulation $\gamma_{\text{mod}} = \frac{\pi}{2}\kappa\delta n_{\text{I}}$ is related to coupling coefficient χ_{ba} by:

$$|\chi_{\text{ba}}| = \frac{4}{\pi}\gamma_{\text{mod}} \tag{1.2}$$

In our nanocavity with dipole presented, the coupled mode equation is written as:

$$\frac{d}{dt}a_{\text{CW}} = -i\omega a_{\text{CW}} - \gamma_{\text{tot}}a_{\text{CW}} + \chi_{\text{ab}}a_{\text{CCW}} + \tau s$$

$$\frac{d}{dt}a_{\text{CCW}} = -i\omega a_{\text{CCW}} - \gamma_{\text{tot}}a_{\text{CCW}} + \chi_{\text{ba}}a_{\text{CW}} + \epsilon s \tag{1.3}$$

Here $a_{\text{CW/CCW}} = \tilde{a}_{\text{CW/CCW}}e^{-i\Omega_d t}$, where Ω_d is the dipole resonant frequency. Note that in the cavity without dipole source, $a_{\text{CW/CCW}}$ is in the form of $\tilde{a}_{\text{CW/CCW}}e^{-i\Omega_\pm t}$, where $\Omega_\pm$ is the eigenfrequency. The total loss include both intrinsic loss γ_{cav} and

loss introduced by the refractive index modulation γ_{mod}. In the simulation, γ_{tot} and γ_{cav} can be determined by the quality factor of the cavity with and without refractive index modulation. And γ_{mod} is then given by $\gamma_{\mathrm{mod}} = \gamma_{\mathrm{tot}} - \gamma_{\mathrm{cav}}$. Combined with Eq. (1.2), we can also obtain $|\chi_{\mathrm{ba}}|$ of the cavity. τ and ϵ is the coupling coefficients of the dipole source to the CW and CCW WGMs, respectively.

Here we take the dipole position as origin since we do not include phase in the source term. Then the Fourier transformation coefficients of the PT symmetric modulation should have a phase term due to the selection of the origin, and the coupling coefficient $\chi_{\mathrm{ab(ba)}}$ is deduced as:

$$\chi_{\mathrm{ab(ba)}} = \kappa(\delta n_{\mathrm{I}} \mp \delta n_{\mathrm{R}}) e^{\mp i2l\varphi_0} \tag{1.4}$$

This means that the relative position of dipole to the refractive index modulation will lead to an initial phase shift to the coupling coefficients. Here $\varphi_0 = 0$ indicates that the dipole is placed at the intersection with refractive index changing from 0 to δn_{R} (Fig. 1b in the main text). Eq. (1.4) is Eq. (4) presented in the main text.

In steady state, using Eq. (1.3), the ratio of $a_{\mathrm{CCW}}/a_{\mathrm{CW}}$ can be deduced as:

$$\frac{a_{\mathrm{CCW}}}{a_{\mathrm{CW}}} = \frac{\epsilon}{\tau} + \frac{\chi_{\mathrm{ba}}}{\gamma_{\mathrm{tot}}} \tag{1.5}$$

Eq. (1.5) is the general case to Eq. (5) in the main text, where the coupling coefficients can be different. When the dipole can only couple to CW mode, $\tau = 1$ and $\epsilon = 0$, and we have $\frac{a_{\mathrm{CCW}}}{a_{\mathrm{CW}}} = \frac{\chi_{\mathrm{ba}}}{\gamma_{\mathrm{tot}}}$. The amplitude of CCW mode is totally originated from the coupling from CW mode to CCW mode χ_{ba}. When the dipole can only couple to CCW mode, $\epsilon = 1$ and $\tau = 0$, the ratio is infinite, and only CCW mode is excited, due to the fact that CCW mode cannot couple to CW mode. When the dipole can couple to both modes but the coupling coefficients are different, the amplitude ratio vanishes at $\chi_{\mathrm{ba}} = -\frac{\epsilon}{\tau}\gamma_{\mathrm{tot}}$. We can still obtain pure CW mode excitation, but the chiral condition is

modified.

As discussed in the main text, when the coupling coefficients are the same, we have:

$$\frac{a_{\mathrm{CCW}}}{a_{\mathrm{CW}}} = 1 + \frac{\kappa(\delta n_{\mathrm{I}} + \delta n_{\mathrm{R}})}{\gamma_{\mathrm{tot}}} e^{i2l\varphi_0} \tag{1.6}$$

Due to the appearance of the phase term $e^{i2l\varphi_0}$, the chirality depends on the location of the dipole. Here the total loss $\gamma_{\mathrm{tot}} = \gamma_{\mathrm{mod}} + \gamma_{\mathrm{cav}}$, and γ_{cav} is the intrinsic cavity loss. For system with balanced gain and loss, $\gamma_{\mathrm{mod}} = 0$. When placing the dipole at the maximum gain point, $\varphi_0 = 0$, we have:

$$\frac{a_{\mathrm{CCW}}}{a_{\mathrm{CW}}} = 1 + \frac{\kappa(\delta n_{\mathrm{I}} + \delta n_{\mathrm{R}})}{\gamma_{\mathrm{cav}}} \tag{1.7}$$

We can see that $a_{\mathrm{CCW}}/a_{\mathrm{CW}}$ is larger than one, most of energy is radiated into the CCW mode. However, the pure CCW mode is achieved only when $\gamma_{\mathrm{cav}} = 0$. In this limit, all the energy of CW mode will finally couple to CCW mode, leading to pure CCW mode excitation. In practical design, $\gamma_{\mathrm{cav}} \neq 0$. Furthermore, for quantum application, we should stick to the passive PT modulation. where $\gamma_{\mathrm{mod}} \neq 0$. Therefore pure CCW mode excitation is unpractical.

Then we turn to the pure CW mode excitation by using the phase cancellation. This can be realized when $\frac{a_{\mathrm{CCW}}}{a_{\mathrm{CW}}}$ equals to zero at $\chi_{\mathrm{ba}} = -\gamma_{\mathrm{tot}}$. In this case, the cavity loss is given by: $\gamma_{\mathrm{cav}} = \gamma_{\mathrm{tot}} - \gamma_{\mathrm{mod}} = \left(1 - \frac{\pi}{4}\right)\gamma_{\mathrm{tot}}$. And $e^{i2l\varphi_0}$ should be equal to -1, thus the dipole emitter should be placed at $\varphi_0 = \frac{\pi}{2l}$, corresponding to the center of the loss region. Here the phase difference between directly radiated and the backscattered CCW waves is π, leading to the destructive interference between them.

Note S2: Detailed full wave simulation description.

For traveling WGM supported by the ring cavity, the wave is propagating along the azimuthal (φ) direction. We assumed that the transverse mode profile at ρ z plane of the propagating WGM is close to the mode propagating in straight waveguide with the same cross section. We calculate the effective index of the propagating mode in straight waveguide (Mode analysis section in RF module, Comsol Multiphysics) to approximate the effective index of the propagating WGM. In the model, InP region with height of 210 nm is sandwiched between silver. The top and bottom of the silver/InP/silver is set to be air layer and silver layer respectively.

The effective index and mode distribution of the simulated result is shown in Fig. S2. The cavities only support plasmonic modes. The plasmonic modes are formed by the coupling between the plasmonic modes supported by the metal–dielectric interface at both sides. They can be classified into symmetric modes and antisymmetric modes, which exhibit same phase or opposite phase at the two sides. With the decrease of the width of the nanocavity, only the fundamental symmetric plasmonic mode can exist. The antisymmetric modes are cut-off at small ring width. Due to the plasmonic signature, electric field component with polarization parallel to the silver surface at the bottom of the cavity is dominated.

In 2D simulation, we can find that adding periodically arrange additional 2.8 nm Ge layer, 2.1 nm Cr /1.3 nm Ge bilayer, 2.7 nm Cr layer and 1.9 nm Ge layer at the bottom of the InP region ($w = 50$ nm, $h = 210$ nm) in the azimuthal direction can realize PT symmetric refractive index modulation ($\delta n_R = \delta n_I = 0.003$) shown in the main text.

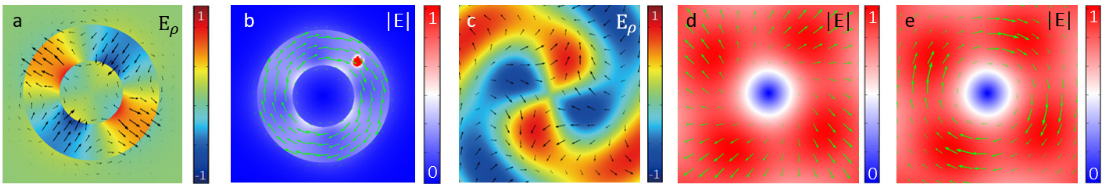

Fig. S1. Single emitter vortex radiation at 900 nm ($l = -2$). E_ρ and $|\mathbf{E}|$ of the single emitter excited field are plotted inside the cavity (a, b) and at a height of 1550 nm above the cavity (c-e). In (c) - (d), the black and green arrows denote polarization and Poynting vector, respectively. In (e), the green arrows denote azimuthal component of Poynting vector. In the simulation, the material parameters is set to be: $n_{\mathrm{Ag}} = 0.0004 + 6.3699i$ for silver at the temperature of 4.5 K, and $n_{\mathrm{GaAs}} = 3.546$ for GaAs. δn_{R} $(= \delta n_{\mathrm{I}})$ is increased from 0.003 to 0.006 since the metal loss is increased. The height of the nanocavity is 120 nm. The width of GaAs region w and r_{in} is set to be 40 nm and 35 nm, respectively.

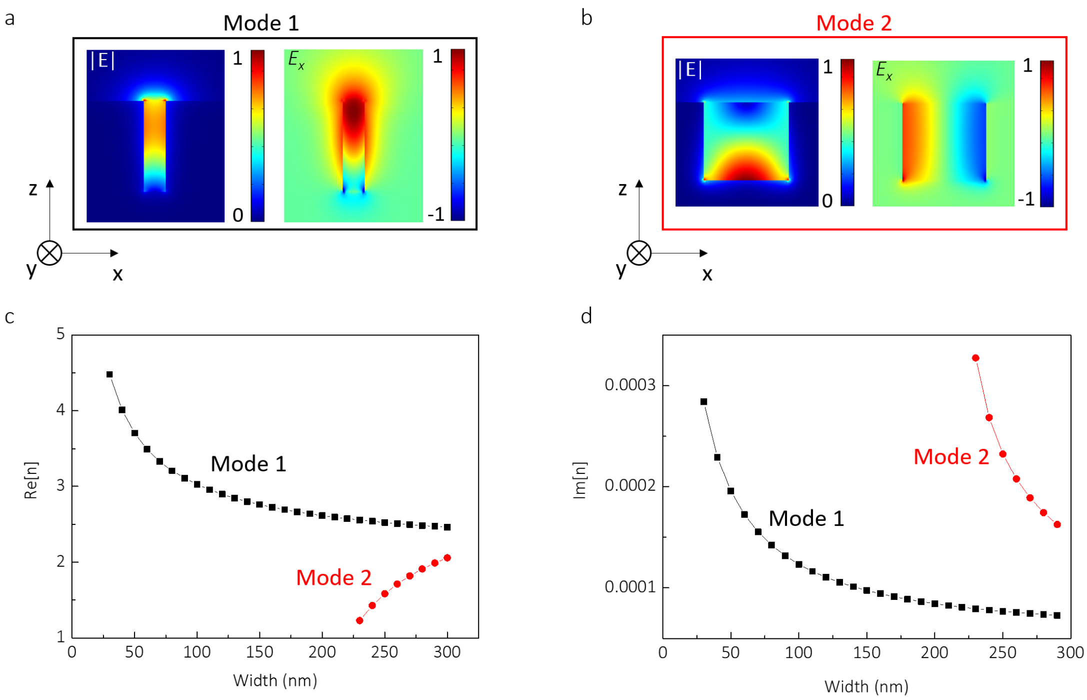

Fig. S2. The 2D mode analysis for calculating the effective refractive index of the chiral plasmonic nanocavity. The real part and imaginary part of the effective refractive index as a function of the width of the ring are presented in the (a) and (b), respectively. The insets of (a) are the corresponding $|\mathbf{E}|$ field (left) and E_x field (right).

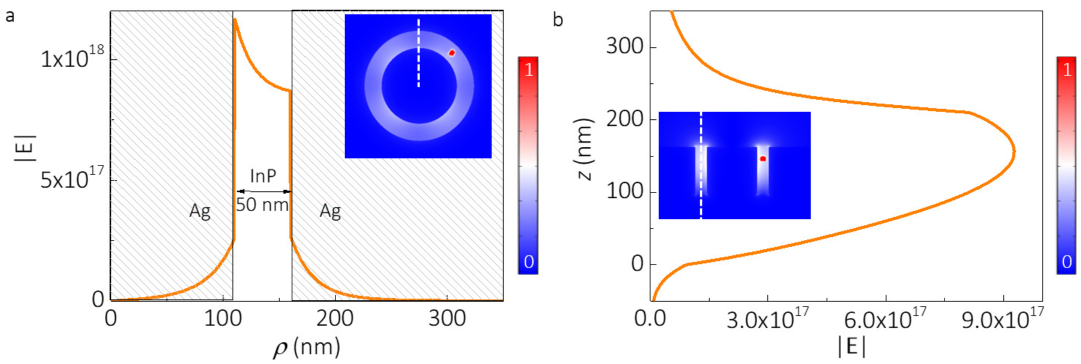

Fig. S3. Strong field confinement of the cavity mode of quantum vortex emitter with $\boldsymbol{\lambda}$ round 1500 nm ($\boldsymbol{l = -2}$). (a) $|\mathbf{E}|$ of the dipole excited field along the radial direction in the transverse cross section inside the chiral plasmonic nanocavity. (b) $|\mathbf{E}|$ of the dipole excited field along z direction in the vertical cross section inside the chiral plasmonic nanocavity.

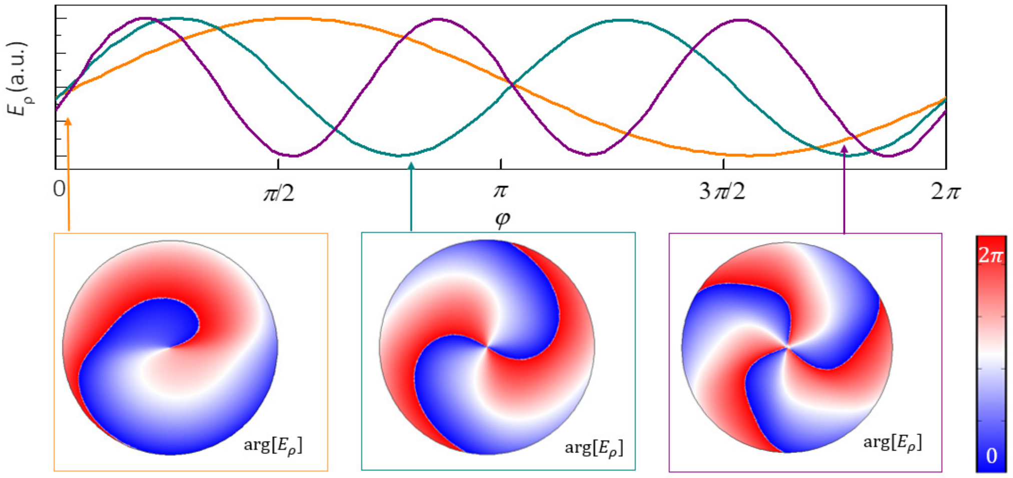

Fig. S4. Field characterization of the cavity mode and its radiation field of quantum vortex emitter with λ round 1500 nm. The upper panel shows that E_ρ (radial component of the electric field) distributions of dipole excited field as a function of φ inside the cavity for $l = -1, -2, -3$. The lower panel shows the phase distribution of E_ρ of the cavity radiation field 1550 nm above the cavity for the three cases.

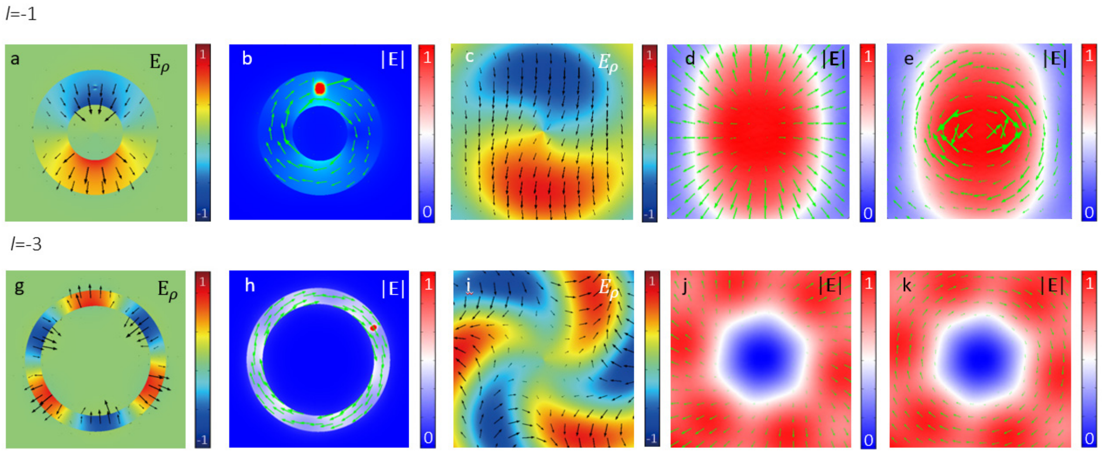

Fig. S5. Vortex beam emission with topological charge $l = -1$ and $l = -3$. E_ρ and $|\mathbf{E}|$ of the single dipole excited field are plotted inside the cavity (a, b for $l = -1$, g, h for $l = -3$) and at a height of 1550 nm above the cavity (c-e for $l = -1$, i-k for $l = -3$). In (a-d) and (g-j), the black and green arrows denote polarization and Poynting vector, respectively. In (e) and k, the green arrows denote azimuthal component of Poynting vector.

Note S3: Numerical calculation of the spontaneous emission coupling factor

Spontaneous emission coupling factor to CW (CCW) WGM is given by $\beta_{\mathrm{CW(CCW)}} = \frac{\Gamma_{\mathrm{CW(CCW)}}}{\Gamma_{\mathrm{CW}}+\Gamma_{\mathrm{CCW}}+\Gamma}$. $\Gamma_{\mathrm{CW(CCW)}}$ is the spontaneous emission rate to CW (CCW) mode and Γ is the emission rate into all other modes. In simulation, β factor is calculated by the ratio of the power radiated into the desired mode to total emission power $P_{\mathrm{mode}}^{\mathrm{dipole}}/P_{\mathrm{total}}^{\mathrm{dipole}}$ of a dipole placing inside the cavity (Fig. S6a).

From Eq. (1.3), we can derive that the ration of $\beta_{\mathrm{CCW}}/\beta_{\mathrm{CW}}$:

$$\frac{\beta_{\mathrm{CW}}}{\beta_{\mathrm{CCW}}} = \frac{|\alpha_{\mathrm{CW}}|^2}{|\alpha_{\mathrm{CCW}}|^2} = \left|1 - \frac{\chi_{\mathrm{ba}}}{i\Delta - \gamma_{\mathrm{tot}}}\right|^2 \tag{3.1}$$

where $\Delta = \Omega_d - \omega$ is the detuning of the dipole resonant frequency with respect to the traveling WGM frequency ω. From Eq. (1.2) and Eq. (1.4), we can derive that:

$$\frac{\chi_{\mathrm{ba}}}{\gamma_{\mathrm{tot}}} = \frac{4}{\pi}\left(1 - \frac{\gamma_{\mathrm{cav}}}{\gamma_{\mathrm{tot}}}\right) e^{i2l\varphi_0} \tag{3.2}$$

$\gamma_{\mathrm{cav}}/\gamma_{\mathrm{tot}}$ can be given by $Q_{\mathrm{tot}}/Q_{\mathrm{cav}}$, where $Q_{\mathrm{cav}} = 1490$ is the Q factor of cavity without refractive index modulation, $Q_{\mathrm{tot}} = 480$ is the Q factor of cavity with refractive index modulation, and thus:

$$\frac{\chi_{\mathrm{ba}}}{\gamma_{\mathrm{tot}}} = 0.86 e^{i2l\varphi_0} \tag{3.3}$$

With this ratio, we can calculated the $\frac{\beta_{\mathrm{CW}}}{\beta_{\mathrm{CCW}}}$ as a function of azimuthal position φ_0 of the dipole (Blue solid line in Fig. S6b) and wavelength (Blue solid line in Fig. S6c). The analytical result matches well with the simulated result (Blue solid dots in Fig. S6b and S6c).

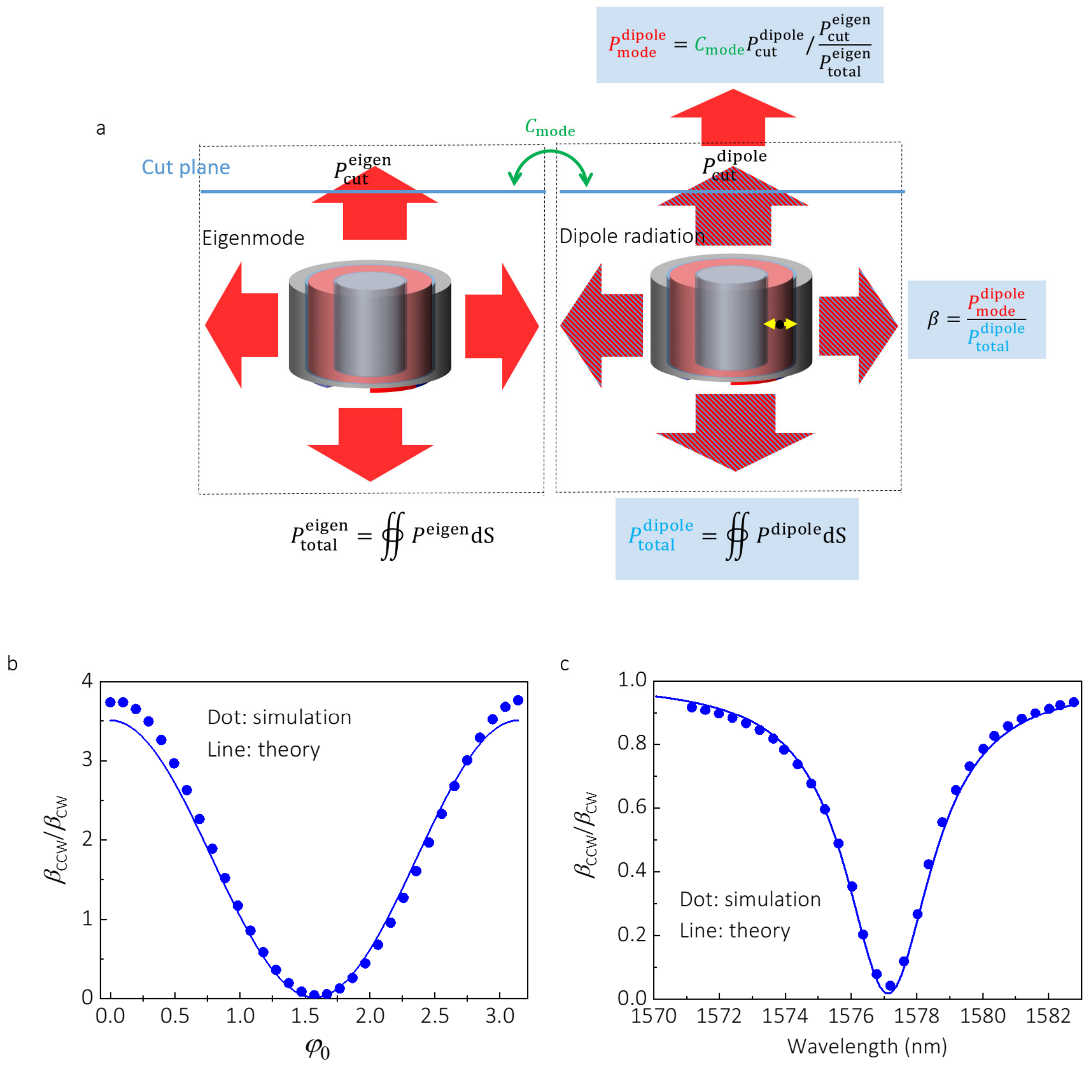

Fig. S6. The calculation of spontaneous emission coupling factor $\boldsymbol{\beta}$. (a) Schematic of the calculation method of β. To calculate β factor, we carried out eigenmode and dipole excitation simulation. In the eigenmode simulation (left panel), we calculate the total power outflow from the cavity P_{total}^{eigen} and power flow perpendicular to a certain cut plane P_{cut}^{eigen} (blue line). In the dipole excitation simulation, we also calculate the total power outflow from the cavity P_{total}^{dipole} and power flow perpendicular to the same cut plane P_{cut}^{dipole} (blue line, right panel). We extracted the field distribution at the cut planes from the two simulation, and obtain their overlap integral coefficient by $\mathrm{C}_{mode} = \left|\iint \left(E_{cut}^{eigen}\right)^{*} E_{cut}^{dipole}\, dxdy\right|^{2}$. The power flow perpendicular to the cut plane

of the desired eigenmode can then be calculated by $C_{\text{mode}}P_{\text{cut}}^{\text{dipole}}$. For a specific eigenmode, the ratio between the power flow perpendicular to the cutplane and the total power flow $\frac{P_{\text{cut}}^{\text{eigen}}}{P_{\text{total}}^{\text{eigen}}}$ is fixed. Therefore, the power of dipole radiated to the desired eigenmode can be obtained by: $P_{\text{mode}}^{\text{dipole}} = C_{\text{mode}}P_{\text{cut}}^{\text{dipole}} / \frac{P_{\text{cut}}^{\text{eigen}}}{P_{\text{total}}^{\text{eigen}}}$. And β is calculated by the ratio of the power radiated into the desired mode ($P_{\text{mode}}^{\text{dipole}}$) to total emission power ($P_{\text{total}}^{\text{dipole}}$) of a dipole placing inside the cavity $\beta = P_{\text{mode}}^{\text{dipole}}/P_{\text{total}}^{\text{dipole}}$. Note that we consider the same polarization for the calculation of C_{mode}, $P_{\text{cut}}^{\text{eigen}}$, $P_{\text{cut}}^{\text{dipole}}$. (b-c) Numerical calculation of spontaneous emission coupling factor of our chiral plasmonic nanocavity. The ratio of the CCW and CW mode as a function of dipole position φ_0 at resonance (b) and wavelength (c). In (b-c), dots and solid line are obtained from full wave simulation and coupled mode theory respectively.
